# Development and validation of a food frequency questionnaire for Japanese athletes (FFQJA)

**DOI:** 10.1186/s12970-021-00433-5

**Published:** 2021-05-10

**Authors:** Kazuko Ishikawa-Takata, Kaori Okamoto, Motoko Taguchi

**Affiliations:** 1 Waseda Institute of Sports Nutrition, 2-579-15 Mikajima, Tokorozawa, Saitama, Japan; 2 Faculty of Applied Biosciences, Tokyo University of Agriculture, 1-1-1 Sakuragaoka, Setagaya, Tokyo, Japan; 3 Graduate School of Sport Sciences, Waseda University, 2-579-15 Mikajima, Tokorozawa, Saitama, Japan; 4 Faculty of Sport Sciences, Waseda University, 2-579-15 Mikajima, Tokorozawa, Saitama, Japan

**Keywords:** Nutritional assessment, Active person, Meal, Dietary record, Food group

## Abstract

**Background:**

Food frequency questionnaires are considered an effective method for assessing habitual dietary intake, but they must be developed or validated with the target population. Portion size, supplement use and food choice are thought to be especially important methodological considerations for assessing athletes’ dietary intake. This study aimed to develop and validate a food frequency questionnaire for Japanese athletes using data from this population.

**Methods:**

We used dietary records from 440 Japanese athletes involved in our previous projects. Food items were analyzed using cumulative percentage contributions and multiple regression analysis, to give a selection of 62 basic food items and four supplemental items. The validity of the questionnaire was evaluated among another 77 Japanese athletes by comparing nutrient intakes assessed using the questionnaire with dietary records. Reproducibility was evaluated by comparing a second questionnaire completed 2–3 weeks later by 36 of the athletes in the validation study. Validity was assessed using crude Spearman’s correlation coefficients (CCs), energy-adjusted CCs, intraclass CCs (ICCs), and Kappa index values. Reproducibility was assessed by CCs, energy-adjusted CCs, and ICCs.

**Results:**

In the validation analysis, the median crude CC for all of the nutrients was 0.407, ranging from 0.222 for dietary fiber to 0.550 for carbohydrate. The median energy-adjusted CC was 0.478, and the median ICC was 0.369. When we divided the athletes into quartiles, 65% (vitamin B_1_) to 86% (iron) of athletes were classified into the same or adjacent categories using the questionnaire and dietary records, with a median Kappa statistic of 0.32. In the reproducibility analysis, the median crude CC between the two completed questionnaires was 0.654, ranging from 0.582 (carbohydrate) to 0.743 (vitamin B_2_). The median energy-adjusted CC was 0.643, and the median ICC was 0.647.

**Conclusions:**

The new 62-item food frequency questionnaire is both reliable and valid and may be useful for assessing food intake in Japanese athletes.

## Background

Adequate nutrient intake is essential for athletes to prevent illness and injury, and optimize athletic performance [1]. The need for nutrition education and/or counseling for athletes is increasing, and dietary assessment is often undertaken routinely [2]. However, accurate nutritional assessment for athletes in practical situations is complex [3, 4].

Food frequency questionnaires (FFQs) are considered an effective method for assessing habitual dietary patterns. However, food lists and portion sizes used in FFQs must be tailored to the target population because dietary habits vary greatly depending on the ethnic, social, and cultural background of participants [5, 6]. The Japanese diet has unique food selection and menu composition characteristics [7]. Athletes also often have very high or low dietary intakes because of high energy expenditure or restrictive dieting to achieve their optimal physique [8]. Burke et al. suggested that, even among athletes, energy intake may vary fivefold, from as little as 6 MJ/day to as much as 30 MJ/day [9]. The Japanese National Health and Nutrition Survey [10] found that median energy intake among Japanese men in their twenties was 2153 kcal, but energy intake in elite Japanese wrestlers varied from 3686 kcal in the training period to 1071 kcal in the weight-in period [11]. Factors affecting food choice also vary between athletes and the general population. Food choices in the general population are affected by several factors including sensory appeal, convenience and social influence [12]. Athletes’ food choice is also affected by factors such as effect on performance [13]. Magkos and Yannakoulia proposed that assessing dietary intake among athletes required special methodological considerations including serving sizes, snacking, water and beverage consumption, supplement use, weight control and seasonality [5]. One study examined the portion size among Japanese non-athletes and found that the median portion size of rice was 78.0 g, with between-person coefficient variation of 27.8% [14]. However, the standard intake of rice among athletes has been measured as 850 g/day [15].

A study comparing energy intake assessed by dietary record and FFQ with fixed portion size suggested significant differences between the two forms of record, with median values of 3121 and 2579 kcal [16]. However, to our knowledge, only two questionnaires have been developed using dietary information from athletes [17, 18]. In other studies, FFQs were developed using dietary databases involving non-athletes, then validated for athletes [3, 19, 20]. However, this approach might result in the foods in the FFQ being different from athletes’ usual food choices. Only two studies have validated FFQs developed for Japanese non-athletes [21, 22] for use with Japanese collegiate athletes [23, 24]. One of these FFQs was developed for middle-aged Japanese people, and includes 138 food and 20 beverage items. Portion size had three levels: smaller than standard size, standard size and larger than standard size. Another 21-question FFQ is commercially available, but its development process is unclear. This questionnaire also has three portion size levels: little, moderate and big.

FFQ results are typically presented as habitual intake of nutrients or foods on a daily basis. However, in education for athletes, the timing between meals and training is currently considered an essential consideration [25]. Although training schedules vary in terms of training periods, during the same training period, most athletes follow a relatively similar daily schedule. Thus, assessing the habitual intake in each meal may provide useful information to inform nutritional advice, depending on the athletes’ training and meal schedule each day. In addition, assessing habitual nutrient intake in each meal could enable the accumulation of evidence regarding timing between meals, training and performance.

In the current study, we developed an FFQ for Japanese athletes (FFQJA) using a nutritional database for athletes, and validated the questionnaire with a sample of Japanese athletes. In addition, we attempted to construct questions to elucidate habitual eating patterns for each meal.

## Methods

### Development of FFQJA

#### Dataset

Pooled data from our previous studies of dietary records were used to develop the FFQJA. This dataset was collected from 440 Japanese athletes (220 men and 220 women) aged 15–28 years. In this study, athletes were defined as people who participated in training sessions to improve their performance and regularly took part in competitions. The median and interquartile ranges for height, body mass, and Body Mass Index (BMI) were 175.0 (171.0, 179.0) cm, 72.4 (67.2, 83.0) kg, and 23.8 (22.3, 26.1) kg/m^2^, respectively, for men, and 162.3 (157.7, 166.9) cm, 57.9 (52.8, 66.1) kg and 22.2 (20.8, 24.3) kg/m^2^, respectively, for women. Participants were engaged in track and field, swimming, soccer, sailing, volleyball, rugby, American football, basketball, handball, baseball, softball, badminton, lacrosse, bicycle racing, judo, gymnastics, rhythmic gymnastics, weightlifting, boxing, fencing, canoeing and cheerleading. This dataset was already de-identified. The ethics committee approved the use of this dataset without personal informed consent. The dataset included 3–7 days of dietary records for each participant, but we randomly selected 1 day of dietary records for each. Medians and interquartile ranges of intake of energy, protein, fat, and carbohydrate were 2834 (2178, 3553) kcal, 94.5 (74.2, 118.8) g, 85.7 (65.0, 112.0) g, and 383.9 (294.9, 508.2) g, respectively.

#### Dietary records

Participants were given a dietary log, a ruler, a scale, and a digital camera. Trained registered dietitians explained the dietary record method, and participants were instructed to record food and drink names, weight, and/or size in detail. Participants were asked to take photographs of all foods and drinks consumed. When processed food/drinks were consumed, participants kept the food labels. Trained registered dietitians confirmed the logs with participants. The registered dietitians then calculated the nutrient intake using nutrient calculation software (WELLNESS 21 ver. 2.84; Top Business System, Okayama, Japan) with the Standard Tables of Food Composition in Japan, 2015 [26]. The nutrient content of processed foods and foods eaten outside the home or purchased to go were calculated using the nutrient databases of the food producers, or divided into ingredients using photographs and logs. Before we used this dataset, one research nutritionist (KO) checked all data to assess whether the calculations had been conducted in a similar way.

#### Concept of FFQJA

To assess changes in dietary intake against the training schedule, we designed the FFQJA to calculate the average intake over a 2-week period. Eleven nutrients were selected: energy, protein, fat, carbohydrate, calcium (Ca), iron (Fe), retinol activity equivalents (RAE), vitamin B_1_, vitamin B_2_, vitamin C, and dietary fiber. These nutrients were selected based on the sufficiency of the nutritive data for all Standard Tables of Food Composition in Japan 2015 [26], food labels of supplements and processed foods, and nutrition recommendations for athletes.

#### Selection of food groups

**Fig. 1:**
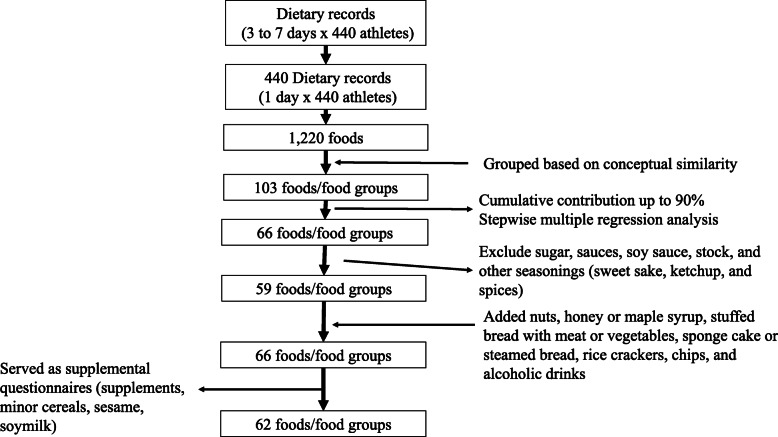
Flow chart of the selection of the foods/food groups list in the food frequency questionnaire for Japanese athletes

Participants recorded 1220 foods in 440 dietary logs (Fig. 1). First, all reported foods, drinks, and supplements were grouped into 103 items based on their conceptual similarity. We defined the percentage contribution of nutrient *k* by food *i* as the arithmetic mean of the individual percentage contribution of nutrient *k* by food (IPC_*jik*_), which was estimated by ${IPC}_{ji k}=\frac{Q_{ji}{D}_{ik}}{\sum \limits_{i=1}^{103}{Q}_{ji}{D}_{ik}}\times 100,$ if $\sum \limits_{i=1}^{103}{Q}_{ji}{D}_{ik}=0$ then IPC*j* = 0 was assumed. The percentage contribution of nutrient k by food i was calculated as $\sum \limits_{j=1}^{440}{IPC}_{jik}/440,$ where j = 1…440 subjects, i = 1…103 food items, k = 1…11 nutrients, Q = grams of foods consumed and D = nutrient content per gram of food. We applied stepwise multiple regression analysis with the overall intake of each nutrient as dependent variables, and 103 grouped items as independent variables. This stepwise regression analysis was used to identify the food items that contributed at least 90% of the between-person variability. Finally, we chose 66 items both by a cumulative percentage contribution of 90% and 0.90 cumulative multiple regression coefficient from the multiple regression analysis with total energy or nutrient intake as dependent variables, and energy or nutrient intake from each item as independent variables. After these selections, we excluded sugar, sauces, soy sauce, stock, and other seasonings (sweet sake, ketchup, and spices) because these items were difficult to report for athletes who did not cook for themselves. Sugar was mainly used as a seasoning during cooking, and not added to drinks (i.e., tea and coffee). Most of the seasoning  intake was included as part of the intake of miso soup, curry or stew, fried dishes, and stir-fried dishes. In addition to the remaining 59 items, we added nuts, honey or maple syrup, bread filled with meat or vegetables, sponge cake or steamed bread, rice crackers, chips, and alcoholic drinks to the questionnaire because these items are often mentioned in nutritional counseling for athletes in Japan [27] (total number of food items = 66). From these 66 items, we presented supplements, minor cereals, sesame, and soymilk as supplemental questions, because these items were thought to be difficult to answer using the same type of question used for the other foods/food groups, and it was difficult to establish portion sizes and/or nutrient databases for the FFQJA. Minor cereals and soy milk were included within the cumulative 90% contribution to Fe and carbohydrate intakes. However, fewer than 30 participants consumed these foods. Sesame contributed to Ca and Fe intake, but the average consumption was only 2.5 g. Establishing average nutrient intake as a nutrient database for the FFQJA for supplements from one portion is difficult because of the wide variation in supplements. Thus, we presented these four items as supplemental questions to prompt participants to list supplements and foods eaten more than once a week, directly reporting the frequency and amount of these foods. As a result, the basic FFQ questionnaire included 62 food items (The food groups were shown in Appendix).

#### Frequency and portion size

The consumption frequencies for 40 items that are usually eaten as meals were reported separately for breakfast, lunch, dinner, and supplement consumption. The consumption frequency for drinks and sweets in a day was also reported. The frequency was classified into four categories: almost every day, three to four times per week, once or twice a week, and never or rarely. The FFQJA assessed food intake over 2 weeks, and “never or rarely” was defined as less than once a week.

The standard portion size was calculated for foods from the dietary records using typical values and/or natural units from the literature. The amount of food consumed was reported via open-ended responses regarding how many times the standard portion size was consumed.

#### Nutrient database for the FFQJA

A nutrient database for the FFQJA was constructed using dietary record data. Using the foods classified into each item in the FFQJA, a weighted nutrient profile of 1–11 foods for each food item was calculated using the Standard Tables of Food Composition in Japan 2015 [26]. The food used in the calculation was selected using the appearance rate or weight contribution for each food item in the dietary record, covering approximately 70% of the total intake of each nutrient from each food group. When participants answered supplemental questions, nutrient content was obtained from the Standard Tables of Food Composition in Japan 2015 [26] or the nutrient databases of the food producers.

### Validation of FFQJA

#### Protocol

**Fig. 2:**
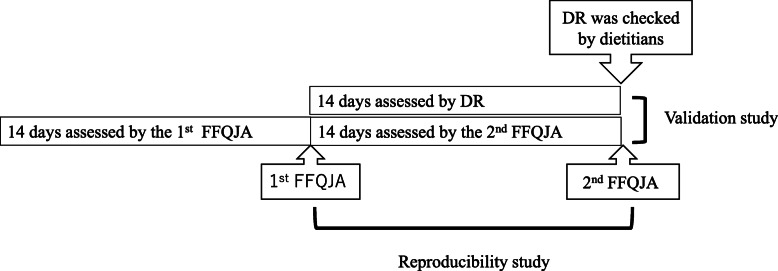
Protocol for development and reproducibility studies

The FFQJA was conducted on the first day of the study. A self-administered dietary record measure was completed over the following 2 weeks (Fig. 2).

#### Participants

We distributed handbills about the study at one high school and one university. We followed this with explanatory meetings for athletes who were interested in this study. A total of 92 athletes aged 15–21 years participated in the validation study. Of these athletes, 14 individuals were excluded before all analyses, of whom four did not complete the 14-day dietary record, one changed his usual diet because he started weight reduction before a competition, and nine were outliers (more or less than three standard deviations from the mean) for energy, protein, fat or carbohydrate intake assessed by the dietary record. This left 78 athletes (41 men and 37 women) in the validation study. Median and interquartile values for height, body mass and BMI were 177.5 (170.2, 182.8) cm, 70.4 (66.7, 75.6) kg, and 22.7 (21.6, 24.0) kg/m^2^, respectively, for men, and 160.0 (157.6, 164.1) cm, 56.7 (52.7, 59.9) kg, and 21.9 (20.7, 23.9) kg/m^2^, respectively, for women. These athletes participated in track and field, ice hockey, weightlifting, bicycle racing, judo, swimming, skiing, skating, table tennis, volleyball, basketball, baseball, lacrosse, and wrestling. All participants and/or their guardians (for athletes younger than 20 years old) were fully informed about all aspects of the study and provided informed consent upon entering the study. This study was approved by the ethics committee of Waseda University (No. 2015–320, 22nd Jan 2015).

#### Dietary records

Dietary records were kept over 14 days, to match the assessment period of the FFQJA. The same method and database used to develop the FFQJA were used in the validation study. However, we did not include the nutrient intakes of supplements. To calculate whole nutrient intake from both dietary records and the FFQJA, we added the nutrient contents from supplements taken by each athlete because the FFQJA did not include average nutrient values for supplements.

### Reproducibility of FFQJA

#### Protocol

The second FFQJA was taken within a week of the 14-day dietary record completion (Fig. 2).

#### Reproducibility of FFQJA

Only one school from the validation study joined the reproducibility study. All 36 athletes from one school completed the second FFQJA.

### Statistical analysis

Data were expressed as median and interquartile range because most variables were not normally distributed.

In the validation study, nutrient intakes and intakes from each food group calculated from dietary records and the FFQJA were compared using crude Spearman’s correlation coefficients (CCs), energy-adjusted CCs, and intraclass CCs (ICCs). The residual method proposed by Willett et al. was used to calculate the energy-adjusted variables [28, 29]. Differences between energy and nutrient intake assessed by dietary record and the FFQJA were compared using the Wilcoxon rank sum test. To examine the effectiveness of ranking, we divided the participants in the validation study into four categories by the intake of each nutrient. We then compared the proportion of athletes who were classified into the same, adjacent, ± 2, and ± 3 categories. Kappa statistics were also calculated. Degree of agreement for the weighted kappa values (K_w_) were characterized as poor (K_w_ <  0.20), acceptable (K_w_ = 0.20–0.59), and good agreement (K_w_ ≥ 0.60) [30].

In the reproducibility study, Spearman’s CCs, energy-adjusted CCs, and ICCs were calculated between the first and second FFQJA. The residual method proposed by Willett et al. was used to calculate the energy-adjusted variables [28, 29].

*P* values less than 0.05 were considered statistically significant. All statistical analyses were conducted using IBM SPSS Statistics ver. 22 (IBM Japan, Ltd., Tokyo, Japan).

## Results

The mean number of foods providing 90% of the cumulative contribution was 28, ranging from 16 for vitamin C to 39 for iron (Table 1). In the stepwise multiple regression analysis, the number of selected items contributing at least 90% of the variance ranged from three items for retinol, vitamin B_1_, and vitamin C to 19 items for iron. After the exclusion of some seasonings and addition of extra food items, cumulative r-squared values for this 66-item questionnaire exceeded 0.9 for all nutrients. In contrast, the set of 62 basic items included in the FFQ had cumulative r-squared values lower than 0.9 for Fe, vitamin B_1_, vitamin B_2_, and dietary fiber.

**Table 1:** Number of selected items and the cumulative contribution to total nutrient intake from selected food items

	Number of items contributing up to 90% cumulative contribution*	Stepwise multiple regression analysis		
		Number of items explaining more than 90% of variance*^2^	Cumulative R^2^ for 66 items*^3^	Cumulative R^2^ for 62 items*^4^
Energy	36	12	0.990	0.980
Protein	29	12	0.995	0.955
Fat	22	11	0.988	0.982
Carbohydrate	29	9	0.990	0.980
Calcium (Ca)	34	16	0.992	0.943
Iron (Fe)	39	19	0.979	0.867
Retinol*^5^	17	3	0.999	0.998
Vitamin B_1_	28	3	0.998	0.450
Vitamin B_2_	30	6	0.991	0.665
Vitamin C	16	3	0.999	0.942
Dietary fiber	27	16	0.975	0.849

*Number of food items to reach cumulative percentage contribution of 90% from 103 foods/food groups

*^2^ Number of food items selected by stepwise multiple regression analysis explaining more than 90% of variance from 103 foods/food groups

*^3^ 66 items were all foods/food groups selected by contribution to 90% cumulative contribution and multiple regression analysis, excluding sugar, sauces, soy sauce, stock, and other seasonings (sweet sake, ketchup, and spices), and including nuts, honey or maple syrup, bread stuffed with meat or vegetables, sponge cake or steamed bread, rice crackers, chips, and alcoholic drinks

*^4^ 62 items were only basic items asked about in the food frequency questionnaire. Supplements, minor cereals, sesame, and soymilk were excluded from the list of 66 items

*^5^ Retinol was calculated as retinol activity equivalents

The median crude CC was 0.407, ranging from 0.222 for dietary fiber to 0.550 for carbohydrate (Table 2). The median energy-adjusted CC was 0.478, ranging from 0.270 for fat to 0.584 for Ca. The median ICC was 0.369, ranging from 0.213 for dietary fiber to 0.470 for carbohydrate. Median percent differences between the dietary record and the FFQJA were within 10% except for − 14.6% for fat and 15.4% for retinol (Table 3). The differences between two methods were significant for energy (*p* = 0.011), protein (*p* = 0.037), fat (*p* = 0.007), carbohydrate (*p* <  0.001), retinol (p = 0.007) and vitamin B_2_ (*p* = 0.044). Table 4 shows the degree of coincidence of nutrient intake. The median proportion was 39% for the same category, and 70% for the same or adjacent categories. When we applied the same or adjacent categories, percentages ranged from 65% for vitamin B_1_ to 86% for Fe.

**Table 2:** Correlation coefficients of energy and nutrient intakes from dietary records and food frequency questionnaire for Japanese athletes. (*n* = 78)

Nutrient intake		DR		FFQJA		Correlation coefficient		
		Median	(Interquartile range)	Median	(Interquartile range)	Crude (95% CI)	Energy-adjusted (95% CI)	ICC (95% CI)
Energy	(kcal)	2560	(2074, 2962)	2502	(2073, 3391)	0.523 (0.324–0.678)	–	0.466 (0.273–0.623)
Protein	(g)	91.1	(72.9, 102.5)	94.5	(73.1, 117.0)	0.445 (0.234–0.616)	0.314 (0.090–0.508)	0.393 (0.188–0.565)
Fat	(g)	80.8	(65.1, 98.2)	73.6	(58.4, 89.3)	0.344 (0.122–0.533)	0.270 (0.043–0.470)	0.338 (0.126–0.521)
Carbohydrate	(g)	347.9	(273.0, 400.4)	374.0	(308.6, 510.9)	0.550 (0.356–0.699)	0.353 (0.132–0.541)	0.470 (0.278–0.626)
Calcium	(mg)	509	(415, 670)	573	(432, 803)	0.379 (0.160–0.562)	0.584 (0.397–0.724)	0.367 (0.207–0.578)
Iron (Fe)	(mg)	8.2	(6.8, 9.9)	8.2	(6.2, 10.4)	0.436 (0.223–0.609)	0.491 (0.397–0.724)	0.409 (0.207–0.578)
Retinol	(ηgRAE)	511	(397, 733)	586	(410, 1094)	0.407 (0.191–0.585)	0.515 (0.286–0.653)	0.351 (0.140–0.531)
Vitamin B_1_	(mg)	1.28	(1.02, 1.58)	1.29	(0.99, 1.77)	0.399 (0.182–0.579)	0.293 (0.286–0.653)	0.369 (0.161–0.546)
Vitamin B_2_	(mg)	1.34	(1.08, 1.60)	1.53	(1.10, 1.89)	0.473 (0.266–0.639)	0.574 (0.314–0.672)	0.433 (0.234–0.597)
Vitamin C	(mg)	109	(77, 154)	126	(71, 170)	0.273 (0.046–0.473)	0.464 (0.067–0.490)	0.256 (0.036–0.451)
Dietary fiber	(g)	13.6	(11.9, 16.7)	13.4	(10.6, 16.6)	0.222 (−0.006–0.428)	0.500 (0.385–0.717)	0.213 (−0.009–0.415)

n = 78

*DR* Dietary records, *FFQJA* Food frequency questionnaire for Japanese athletes, *RAE* retinol activity equivalents

Crude correlation coefficient: Spearman’s correlation coefficients

Energy-adjusted correlation coefficients were calculated using an energy density method

*ICC* intraclass correlation coefficients

95% CI: 95% confidence interval

**Table 3:** Difference and percent difference of energy and nutrient intakes between values from dietary records and food frequency questionnaires

Nutrient intake	Difference	Percent difference (%)	p
Energy	185 (− 329, 808)kcal	9.3 (−14.6, 32.3)	0.011
Protein	7.1 (−13.3, 27.0)g	7.2 (−12.8, 41.8)	0.037
Fat	−12.4 (−24.6, 11.5)g	−14.6 (−29.8, 11.8)	0.007
Carbohydrate	59.7 (−10.3, 141.1)g	5.8 (−1.0, 12.9)	< 0.001
Calcium	40 (−121, 188)mg	7.9 (−20.5, 28.3)	0.175
Iron (Fe)	−0.3 (−1.8, 2.3) mg	−2.7 (− 20.9, 32.2)	0.751
Retinol	67 (−112, 459) ηgRAE	15.4 (−22.7, 82.5)	0.007
Vitamin B_1_	0.04 (−0.22, 0.45)mg	2.4 (−16.5, 33.1)	0.496
Vitamin B_2_	0.09 (−0.20, 0.42)mg	4.9 (−14.4, 26.9)	0.044
Vitamin C	4 (−39, 41)mg	5.7 (−34.6, 40.5)	0.672
Dietary fiber	−0.7 (−4.8, 2.4)mg	−5.5 (−32.3, 24.4)	0.220

Values are shown as median and interquartile range

P: Wilcoxon rank sum test to compare energy and nutrient intake from dietary records and food frequency questionnaires

n = 78

**Table 4:** Comparison of energy and nutrient intakes from dietary records and food frequency questionnaires for Japanese athletes based on joint classification by quintiles

Nutrient intake	Joint classification by quintile (%)				Weighted kappa statistics	
	Same category	Adjacent category	± 2 categories	± 3 categories	Kappa statistics	95% confidence interval
Energy	49	30	18	4	0.38	0.22–0.54
Protein	36	43	13	9	0.23	0.07–0.40
Fat	32	43	17	9	0.17	0.01–0.33
Carbohydrate	39	38	18	6	0.25	0.09–0.42
Calcium	43	42	14	3	0.38	0.23–0.52
Iron (Fe)	32	53	9	6	0.27	0.13–0.42
Retinol	49	31	18	3	0.40	0.24–0.56
Vitamin B_1_	30	35	32	4	0.11	−0.06–0.27
Vitamin B_2_	43	42	14	3	0.38	0.23–0.52
Vitamin C	36	48	13	4	0.32	0.17–0.46
Dietary fiber	43	42	12	5	0.36	0.21–0.51

n = 78

When we compared the validity of food intake, the median crude CC was 0.336, ranging from − 0.147 for oil to 0.682 for cereals (Table 5). Sugar and oil showed negative crude CC. The median energy-adjusted CC was 0.345, ranging from − 0.271 for oil to 0.532 for dairy products. The median ICC was 0.217, ranging from − 0.517 for oil to 0.609 for cereals.

**Table 5:** Comparison of intakes of each food group from dietary records and food frequency questionnaires for Japanese athletes

	DR (g/day)		FFQJA (g/day)		Correlation coefficient		
	Median	(Interquartile range)	Median	(Interquartile range)	Crude (95% CI)	Energy-adjusted (95% CI)	ICC (95% CI)
Cereals	596.4	(479.6, 818.0)	680.8	(526.4, 938.6)	0.682 (0.521–0.796)	0.431 (0.218–0.60)	0.609 (0.448–0.732)
Potatoes	34.4	(18.9, 56.4)	12.6	(6.3, 18.9)	0.207 (−0.022–0.415)	0.299 (0.074–0.495)	0.121 (−0.103–0.334)
Sugar	5.3	(3.1, 8.0)	0.0	(0.0, 4.2)	−0.005 (− 0.230–0.221)	− 0.034 (− 0.258–0.193)	0.076 (− 0.147–0.293)
Nuts and seeds	1.0	(0.2, 2.7)	0.0	(0.0, 1.1)	0.403 (0.186–0.582)	0.413 (0.198–0.590)	0.335 (0.123–0.518)
Green and yellow vegetables	78.7	(48.2, 123.8)	58.2	(32.6, 90.8)	0.385 (0.167–0.567)	0.431 (0.218–0.605)	0.302 (0.086–0.490)
Other vegetables	129.6	(101.4, 169.0)	105.6	(60.1, 159.2)	0.112 (−0.117–0.330)	0.152 (− 0.077–0.366)	− 0.024 (− 0.244–0.198)
Fruits	117.5	(54.3, 247.8)	131.9	(77.7, 302.7)	0.478 (0.271–0.643)	0.513 (0.312–0.670)	0.432 (0.233–0.596)
Mushrooms	6.4	(2.9, 11.8)	4.2	(2.1, 6.4)	0.424 (0.210–0.599)	0.488 (0.283–0.650)	0.221 (0.000–0.422)
Seaweed	2.4	(1.2, 4.7)	0.4	(0.2, 1.1)	0.123 (−0.106–0.340)	0.141 (−0.088–0.356)	0.116 (−0.108–0.329)
Beans	30.1	(13.3, 50.8)	25.6	(14.3, 43.8)	0.358 (0.137–0.545)	0.461 (0.252–0.629)	0.357 (0.147–0.536)
Fish	45.0	(26.0, 61.9)	39.3	(27.0, 63.9)	0.177 (−0.052–0.389)	0.180 (− 0.049–0.391)	0.146 (− 0.078–0.356)
Meat	151.9	(101.4, 202.5)	132.4	(97.5, 215.9)	0.421 (0.207–0.597)	0.415 (0.200–0.592)	0.379 (0.172–0.554)
Eggs	43.6	(28.0, 64.6)	42.5	(25.4, 64.0)	0.378 (0.159–0.562)	0.329 (0.106–0.521)	0.406 (0.203–0.575)
Dairy products	133.1	(75.9, 216.4)	147.8	(65.0, 258.1)	0.506 (0.304–0.665)	0.532 (0.106–0.521)	0.495 (0.308–0.646)
Oil	15.0	(10.6, 21.5)	19.3	(12.8, 27.7)	−0.147 (−0.362–0.082)	−0.271 (−0.047–[− 0.044])	− 0.157 (− 0.365–0.067)
Sweets	35.7	(12.9, 49.0)	55.7	(29.6, 89.9)	0.280 (0.054–0.479)	0.361 (0.140–.547)	0.179 (−−0.044–0.385)
Luxury drinks	82.6	(32.9, 174.9)	215.0	(52.5, 564.4)	0.313 (0.089–0.507)	0.276 (0.050–0.475)	0.212 (−0.009–0.414)
Seasonings	246.7	(196.6, 314.0)	27.3	(18.2, 40.1)	0.306 (0.081–0.501)	0.251 (0.023–0.454)	0.104 (−0.120–0.318)

n = 78

*DR* Dietary records, *FFQJA* Food frequency questionnaire for Japanese athletes

Crude correlation coefficient: Spearman’s correlation coefficients

Energy-adjusted correlation coefficients were calculated using an energy density method

*ICC* Intraclass correlation coefficients

95% CI: 95% confidence interval

The median crude CC, energy-adjusted CC, and ICC for nutrient intake between the first and second FFQJA were 0.654, 0.614, and 0.643 (Table 6). The lowest ICC was 0.588 for carbohydrate, and the highest was 0.755 for dietary fiber. The median crude CC, energy-adjusted CC, and ICC values for food intake were 0.553, 0.601, and 0.664 (Table 7). The lowest ICC was 0.417 for meat, and the highest was 0.903 for sugar.

**Table 6:** Reproducibility of food frequency questionnaire for energy and nutrient intakes

Nutrient intakes		1st FFQJA		2nd FFQJA		Correlation coefficient		
		Median	(Interquartile range)	Median	(Interquartile range)	Crude (95% CI)	Energy-adjusted (95% CI)	ICC (95% CI)
Energy	(kcal)	2503	(2065, 3416)	2536	(2051, 3098)	0.597 (0.313–0.783)	–	0.619 (0.373–0.784)
Protein	(g)	91.7	(66.8, 116.0)	95.1	(71.2, 111.9)	0.663 (0.403–0.824)	0.644 (0.376–0.812)	0.623 (0.379–0.787)
Fat	(g)	73.6	(58.4, 94.0)	69.5	(50.8, 86.8)	0.689 (0.440–0.840)	0.631 (0.358–0.804)	0.606 (0.354–0.775)
Carbohydrate	(g)	402.2	(311.5, 512.5)	362.5	(291.4, 439.9)	0.582 (0.293–0.773)	0.563 (0.269–0.761)	0.588 (0.330–0.764)
Calcium	(mg)	588	(422, 811)	488	(412, 783)	0.604 (0.322–0.788)	0.421 (0.098–0.664)	0.634 (0.406–0.799)
Iron (Fe)	(mg)	7.9	(5.9, 10.6)	8.4	(6.1, 9.8)	0.721 (0.487–0.858)	0.654 (0.390–0.819)	0.735 (0.542–0.854)
Retinol^a^	(ηgRAE)	540	(362, 960)	581	(422, 990)	0.633 (0.361–0.806)	0.597 (0.390–0.819)	0.689 (0.474–0.827)
Vitamin B_1_	(mg)	1.30	(0.89, 1.79)	1.17	(1.01, 1.67)	0.654 (0.390–0.819)	0.577 (0.287–0.770)	0.634 (0.394–0.793)
Vitamin B_2_	(mg)	1.48	(1.02, 1.85)	1.35	(1.03, 1.92)	0.743 (0.521–0.871)	0.562 (0.267–0.761)	0.721 (0.521–0.846)
Vitamin C	(mg)	103	(59, 174)	122	(68, 159)	0.737 (0.521–0.868)	0.745 (0.524–0.872)	0.752 (0.569–0.864)
Dietary fiber	(g)	12.3	(10.0, 16.4)	11.5	(9.1, 16.0)	0.654 (0.390–0.819)	0.679 (0.425–0.834)	0.755 (0.574–0.866)

*n* = 36

*FFQJA* Food frequency questionnaire for Japanese athletes

Crude correlation coefficient: Spearman’s correlation coefficients

Energy-adjusted correlation coefficients were calculated using an energy density method

*ICC* Intraclass correlation coefficients

^a^ Retinol was calculated as retinol activity equivalents (RAE: retinol activity equivalents)

95% CI: 95% confidence interval

**Table 7:** Reproducibility of food frequency questionnaire for intakes from each food group

	1st FFQJA (g/day)		2nd FFQJA (g/day)		Correlation coefficient		
	Median	(Interquartile range)	Median	(Interquartile range)	Crude (95% CI)	Energy-adjusted (95% CI)	ICC (95% CI)
Cereals	801.5	(626.9, 952.3)	676.8	(552.8, 855.9)	0.560 (0.265–0.759)	0.595 (0.310–0.782)	0.533 (0.257–0.729)
Potatoes	12.6	(6.3, 22.2)	12.6	(6.3, 19.1)	0.453 (0.135–0.687)	0.447 (0.128–0.682)	0.425 (0.122–0.656)
Sugar	0.0	(0.0, 1.1)	0.0	(0.0, 2.1)	0.859 (0.714–0.933)	0.855 (0.707–0.931)	0.903 (0.819–0.949)
Nuts and seeds	0.0	(0.0, 1.1)	0.0	(0.0, 1.1)	0.405 (0.080–0.652)	0.421 (0.098–0.664)	0.845 (0.719–0.917)
Green and yellow vegetables	48.5	(24.7, 78.0)	57.3	(31.8, 92.7)	0.693 (0.446–0.842)	0.693 (0.446–0.842)	0.826 (0.688–0.907)
Other vegetables	103.1	(56.2, 150.8)	109.2	(61.8, 165.8)	0.546 (0.247–0.750)	0.607 (0.326–0.789)	0.718 (0.517–0.844)
Fruits	143.8	(67.2, 316.4)	170.0	(96.5, 254.3)	0.767 (0.558–0.884)	0.768 (0.560–0.885)	0.583 (0.323–0.761)
Mushrooms	4.2	(2.1, 8.6)	2.1	(2.1, 6.4)	0.508 (0.200–0.725)	0.507 (0.199–0.724)	0.657 (0.427–0.808)
Seaweed	0.4	(0.2, 1.1)	0.4	(0.2, 1.0)	0.341 (0.009–0.605)	0.428 (0.106–0.669)	0.801 (0.647–0.892)
Beans	25.6	(17.0, 44.5)	27.3	(16.8, 43.3)	0.525 (0.221–0.736)	0.524 (0.220–0.735)	0.629 (0.387–0.790)
Fish	37.8	(26.3, 56.5)	39.9	(21.0, 67.8)	0.546 (0.247–0.750)	0.593 (0.308–0.781)	0.505 (0.221–0.710)
Meat	127.5	(99.8, 192.7)	159.5	(106.0, 214.9)	0.500 (0.190–0.719)	0.473 (0.158–0.701)	0.417 (0.112–0.651)
Eggs	42.0	(21.0, 53.5)	32.0	(21.0, 55.9)	0.807 (0.624–0.906)	0.815 (0.637–0.910)	0.750 (0.565–0.863)
Dairy products	177.7	(82.4, 302.8)	133.3	(81.4, 241.3)	0.624 (0.349–0.800)	0.666 (0.407–0.826)	0.673 (0.450–0.817)
Oil	21.2	(13.8, 26.7)	16.8	(10.6, 21.3)	0.546 (0.247–0.750)	0.631 (0.358–0.804)	0.520 (0.239–0.720)
Sweets	54.6	(22.3, 90.6)	47.9	(20.5, 77.2)	0.782 (0.583–0.893)	0.815 (0.637–0.910)	0.664 (0.437–0.812)
Luxury drinks	283.5	(52.5, 536.3)	150.5	(78.8, 459.0)	0.707 (0.466–0.850)	0.587 (0.300–0.777)	0.664 (0.437–0.812)
Seasonings	29.0	(20.0, 45.5)	28.0	(16.8, 42.6)	0.669 (0.411–0.828)	0.729 (0.499–0.863)	0.692 (0.478–0.829)

n = 36

*FFQJA* Food frequency questionnaire for Japanese athletes

Crude correlation coefficient: Spearman’s correlation coefficients

Energy-adjusted correlation coefficients were calculated using an energy density method

*ICC* Intraclass correlation coefficients

95% CI: 95% confidence interval

## Discussion

In this study, we developed and validated a food frequency questionnaire for Japanese athletes engaged in a range of sports. The FFQJA is the first FFQ developed using dietary record data of Japanese athletes, and validated among the same population. In total, 62 food items and four supplemental free questions covered energy and the intake of 10 nutrients. The results showed moderate validity for all nutrients, and quintiles suggested good joint classification.

We designed the FFQJA to be able to assess nutrient intake from each meal. FFQs are excellent tools to assess habitual nutritional intake. However, most of the FFQs that are currently available can only assess mean daily intake during a target period. The FFQJA requires us to make an additional assessment of the time of each meal and training, but it enables us to discuss the timing between nutrient intake and training more easily. Burke pointed out that the time of consumption over a day or in relation to exercise is one of the features of interest for athletes’ dietary intake [31]. The FFQJA can therefore assess the relationship between exercise and meals, but it asks about supplemental meals other than breakfast, lunch, and dinner as a single category (snacks), so cannot be used to fully assess supplemental intake during, just before, and after training.

The FFQJA includes 62 basic food items. We asked participants to report intakes of supplements, minor cereals, sesame, and soy milk as supplemental questions because minor cereals, sesame and soymilk contributed strongly to the nutrient intake, but the number of participants who ate these foods, and the average amount eaten at one time, were very small. We also thought it was not practical to apply the standard nutrient content for supplements. The Japanese diet has unique characteristics [32], and athletes often make specific food choices to improve their performance or physique [33]. The use of nutritional data from a similar population to the target population is essential for developing FFQs [34]. This study used a database of Japanese athletes’ dietary intake. The selected items differed from those reported in an FFQ for Brazilian athletes [17]. In addition, a previous review [34] indicated that the number of food items in FFQs ranged from five to 350, with a median of 79. The number of food items included in this study was close to this median value. When we included 66 items, including the supplemental questions, all cumulative r-squared values were higher than 0.9. These values were higher than those reported in previous studies [17, 35].

In a validation study, we compared the FFQJA with self-administered dietary records. A previous review [34] indicated that an interviewer-administered questionnaire gave higher CCs than a self-administered questionnaire. In contrast, previous studies of FFQs for Japanese populations have often used self-administered dietary records rather than either interviewer-administered dietary records or 24-h recall [36]. In Japan, self-administered dietary records are often used to assess dietary intake [36]. This is because most Japanese people can understand them and are educated enough to be able to complete them on their own. The crude CCs in this study were higher than the median value of other FFQs for Japanese populations, except for fat, Ca, vitamin C, and dietary fiber [36]. Energy-adjusted CCs were better than those reported in a previous study of Japanese athletes using an FFQ developed for non-athletes [23, 24]. This improvement was caused by the selection of food items using assessment data from Japanese athletes and open-ended questions about portion size. Previous studies used FFQs developed for non-athletes, with fixed portion sizes. For non-athletes, between-person variation in portion size tends to be smaller than the variation in frequency [37]. However, athletes may show greater variation in portion size.

We believe that the FFQJA is a valuable tool for assessing nutrient intake in a group, or as a ranking tool for assessing the nutrient intake for athletes. FFQs are often evaluated as a ranking tool for nutrient intake rather than assessing individual nutrient intake [38]. In this study, all nutrients showed a higher proportion of the same or adjacent categories by joint cross-classification, compared with previous studies of Japanese athletes [23, 24].

Even after energy-adjustment, CCs for fat and vitamin B_1_ were weak between dietary records and FFQJA. We speculate that the low CC for fat may have been partially caused by inaccurate estimation of fat intake from deep-fried or stir-fried dishes. In future studies, we need to re-examine the nutrient data for these food types. For vitamin B_1_, stepwise multiple regression analysis selected the smallest number of items (15 items) for vitamin B_1_, so the sources of vitamin B_1_ are thought to be limited. Small differences in food eaten during the study period may have affected vitamin B_1_ intake because the study period for the dietary records and FFQJA was different in this study. However, the study data showed better CCs compared with other studies [39].

Crude CCs for each food group were similar or lower than those reported in studies using other FFQs for Japanese populations [36]. Crude and energy-adjusted CCs for sugar and oil showed negative values. The median intake of seasonings showed a substantial difference between dietary records and the FFQJA. In the FFQJA, participants were asked to report their use of mayonnaise and dressing, and intake of deep-fried food, stir-fried food, and curry and/or stew, to assess their consumption of oil. In contrast, in the dietary records, dietitians asked about or estimated all oils and seasonings consumed. Asian food contains many seasonings, which contribute strongly to nutrient intake [40]. However, we thought that assessing seasoning intake using an FFQ was difficult, especially for participants who did not cook for themselves.

The reproducibility of the FFQJA was excellent, both for nutrients and food-group intake, compared with previous studies using FFQs for Japanese populations [36]. The reproducibility of the proposed measure was relatively good even compared with a previous study of Japanese athletes [23]. The structure of the new questionnaire, reflecting the structure of Japanese meals, including staple foods and main and side dishes, appeared to be easy for athletes to follow.

This study had several limitations. First, participants were recruited from one area in Japan, although they came from various regions. Their habitual eating patterns may therefore not be fully representative of Japanese athletes in all regions. Second, the assessment periods were different for dietary records and the FFQJA in the validation study. We administered the FFQJA on the first day of the survey, and the dietary records over the following 2 weeks. The difference in food eaten during each period may therefore have affected the results of the validation study. Third, we excluded 14 participants from the athletes recruited for the validation study, a loss of nearly 15%. This was mainly because of a lack of 14-day dietary records, or outliers in intake of nutrients assessed by the dietary records. This may have affected the results of the validation study. However, we checked the dietary records of the excluded athletes, revealing that their records were not appropriate for the analysis. Fourth, one of the features of the FFQJA is assessing each meal, but we only validated the daily nutrient and food intakes. Future studies will be needed to validate the intake for each meal and assess the usefulness of information about each meal for nutritional intervention. Furthermore, we did not assess the time of each meal. To examine the relationship between timing of meals and training sessions, it will be necessary to add questions to obtain these data in future. In addition, we only divided the meal category into three meals and “other”. However, this division might not have been sufficiently detailed. Finally, biomarkers were not used to validate the FFQJA. A previous review of the validity of dietary assessment in athletes suggested that energy intake is typically under-estimated by 19% compared with energy expenditure measured using the doubly labeled water method [3]. We plan to conduct an additional validation study using biomarkers in the future.

## Conclusion

In this study, we successfully developed and validated a new food frequency questionnaire for Japanese athletes. We used a database of food intake for athletes, and validated the measure with a range of athletes as respondents. This study did not use biomarkers for the validation study, or validate the intake for each meal. Future research should address these issues in more detail. Despite these limitations, however, the structure of the proposed questionnaire for assessing each meal will be useful for future education/counseling for athletes, and informing the development of new questionnaires in other countries.

## Data Availability

The datasets generated and/or analyzed during this study are not publicly available because our ethical approval did not include the use of these data by other researchers. The final version of the FFQJA is available from the corresponding author on reasonable request.

## Appendix

### List of food groups in the food frequency questionnaire for Japanese athletes (FFQJA)

#### Basic food group

1. Rice.
2. Miso soup.
3. Bread included in a cooked dish (e.g., bread with meat or curry, sandwich, hot dog).
4. Bread with sweet toppings (e.g., bread with jam, cream, or chocolate).
5. Other simple bread including sliced bread or baguette.
6. Use of butter with bread.
7. Use of jam, honey, or maple syrup with bread or yoghurt.
8. Cupped ramen and instant ramen.
9. Japanese noodles, pasta, and Chinese noodles.
10. Cereals.
11. Ham, bacon, and sausage.
12. Fillet or white meat (meat contains less fat).
13. Liver and other internal organs of cows, chicken or pigs.
14. Other beef, chicken, pork or other meat (i.e., excluding products included in questions 11 to 13).
15. Do you remove skins or fatty part of meat?
16. Fish.
17. Canned tuna.
18. Squid, shrimp, octopus, and crab.
19. Shellfish (e.g., clam, oyster).
20. Boiled fish paste and fish sausage.
21. Small fish and fish eggs (roe).
22. Eggs.
23. Tofu (bean-curd) and other processed bean curd.
24. Natto (fermented soybeans).
25. Other products made from soy bean.
26. Potatoes (including taro, yam).
27. Tomatoes.
28. Pumpkin.
29. Carrots.
30. Spinach and other green leaves.
31. Other dark colored vegetables (e.g., green pepper, broccoli).
32. Burdock, lotus root.
33. Light colored leaves (e.g., lettuce, cabbage).
34. Light colored vegetables (e.g., cucumber, radish, onion, eggplant).
35. Mushrooms.
36. Seaweed.
37. Milk.
38. Yoghurt.
39. Cheese.
40. Citrus fruits (e.g., orange, grapefruit).
41. Bananas.
42. Other fruits (e.g., strawberry, kiwi fruit, pineapple, apple).
43. Deep fried dishes.
44. Sautéed dishes.
45. Curry, stew.
46. Mayonnaise, dressing.
47. Vegetable juice.
48. Fruit juice (100% only) (e.g., orange, apple, grapefruit).
49. Sports drink.
50. Other sweetened drink (e.g., soda, tea or coffee with sugar).
51. Alcoholic drink.
52. Nuts (e.g., almond, walnut, peanut).
53. Pudding, jelly.
54. Ice cream.
55. Sponge cake, steamed puddings.
56. Cake (e.g., decorated cake, doughnut).
57. Japanese sweets.
58. Rice crackers.
59. Cookies.
60. Potato chips, popcorn, and other snacks.
61. Chocolate.
62. Candy, gum.

#### Supplemental questions

1. Supplements.
2. Minor cereals.
3. Sesame.
4. Soymilk.
  Free responses can include other food eaten more than once a week.

## Abbreviations

FFQ: Food frequency questionnaire
FFQJA: Food frequency questionnaire for Japanese athletes
BMI: Body Mass Index
DR: Dietary record
CC: Correlation coefficient
ICC: Intraclass correlation coefficient
RAE: Retinol activity equivalents
